# From Toxin to Therapy: Biomedical Applications of Bee Venom in Cancer, Diabetes, and Neurodegenerative Disorders

**DOI:** 10.3390/ijms27114661

**Published:** 2026-05-22

**Authors:** Kassyane de Amorim Lourenço, Mariana Valenhes dos Santos, Adriano C. Araujo, Elen L. Guiguer, Rui Curi, Márcia Gabaldi Rocha, Everton Salgado Monteiro, José Luiz Yanaguizawa Junior, Tânia Pithon-Curi, Karina Quesada, Luiz Carlos de Abreu, Camila de Oliveira Marcondes, Sandra Maria Barbalho, Vitor E. Valenti, Maria Angélica Miglino

**Affiliations:** 1 Department of Biochemistry and Pharmacology, School of Medicine, University of Marília (UNIMAR), Marília 17525-902, SP, Brazil; kassyane.a.lourenco@hotmail.com (K.d.A.L.); mvalenhes@gmail.com (M.V.d.S.);; 2 Postgraduate Program in Structural and Functional Interactions in Rehabilitation, University of Marília (UNIMAR), Marília 17525-902, SP, Brazilmmiglino@outlook.com (M.A.M.); 3 Department of Biochemistry and Nutrition, School of Food and Technology of Marília (FATEC), Marília 17500-000, SP, Brazil; 4 Butantan Institute, São Paulo 05503-900, SP, Brazil; 5 Bioallergy-Pesquisa e Inovação em Alergia, Faculdade de Medicina da Universidade de São Paulo, FMUSP, São Paulo 01246-903, SP, Brazil; 6 School of Medicine, Faculty of Education & Health Sciences, University of Limerick, V94 T9PX Limerick, Ireland; 7 Department of Program in Public Health and Nutrition and Health, Federal University of Espírito Santo (UFES), Vitória 29075-910, ES, Brazil; 8 UNIMAR Charity Hospital, Universidade de Marília (UNIMAR), Marília 17525-902, SP, Brazil; 9 Faculty of Philosophy and Sciences, University Estadual Paulista (UNESP), Marília 17525-900, SP, Brazil

**Keywords:** bee venom, cancer, diabetes, neurological diseases, apamin, melittin, adolapin, phospholipase A2, inflammation, oxidative stress, pain

## Abstract

Apitherapy is a complementary therapeutic approach based on the use of bee-derived products, particularly bee venom (BV), also known as apitoxin. Bee venom is a complex mixture of biologically active compounds, including peptides, enzymes, and biogenic amines, that exhibit diverse pharmacological activities. Major bioactive constituents such as melittin, apamin, adolapin, and phospholipase A2 have attracted increasing scientific interest due to their anti-inflammatory, antioxidant, antimicrobial, analgesic, and immunomodulatory properties. This review provides a comprehensive overview of the biological effects and therapeutic potential of bee venom in the management of chronic diseases, particularly diabetes, cancer, and neurological disorders. Evidence from experimental and clinical studies suggests that BV and its components can modulate multiple molecular pathways associated with oxidative stress, inflammation, apoptosis, and immune responses. These mechanisms contribute to potential benefits in glycemic control, tumor suppression, neuroprotection, and pain management. Additionally, bee venom has been investigated for its capacity to influence signaling pathways involved in cellular proliferation and survival, highlighting its potential as a complementary strategy in the treatment of complex diseases such as neurodegenerative disorders, including Parkinson’s and Alzheimer’s diseases. Despite these promising therapeutic effects, the clinical use of BV remains limited due to safety concerns, particularly the risk of allergic reactions, systemic toxicity, and anaphylaxis. Recent advances in drug delivery systems and nanotechnology may help improve the safety and efficacy of BV-based therapies by enabling targeted delivery and controlled dosing. Overall, bee venom represents a promising source of bioactive compounds with potential applications in translational and integrative medicine; however, further well-designed clinical trials and mechanistic studies are necessary to establish its safety, efficacy, and long-term therapeutic value.

## 1. Introduction

Apitherapy is a branch of complementary medicine that is based on the use of bee products, mainly bee venom (apitoxin), for the treatment of various human diseases [1,2,3,4]. Honeybee venom (BV) is secreted by a gland located in the abdomen of the bee *Apis mellifera*. It is an odorless and colorless liquid with an acidic pH, which bees often use as a defense against predators [5]. Beekeeping products have been used since ancient times, and their therapeutic efficacy is mentioned in the Holy Quran and the Bible [6]. The medicinal use of apitoxin dates back to ancient Egypt and Greece and has been practiced in China for 3000–5000 years [7].

On the market, it is possible to find BV in different forms, such as injections, ointments, tablets, creams, natural bee stings, and pure liquid venom. Furthermore, some specific laboratories can supply their components in isolation for medicinal and scientific purposes [8,9,10,11]. As a toxin, BV can cause mild to severe clinical effects, depending mainly on the dose administered. Symptoms may include allergic reactions, local inflammatory reactions, anaphylactic shock, and systemic toxicity. The estimated lethal dose of bee venom in humans has been reported to be approximately 2.8 mg/kg under conditions of massive envenomation; however, toxicity is highly variable and depends on several factors (route of exposure, number of stings, age, body weight, allergic predisposition, and individual immune sensitivity). Importantly, severe systemic reactions and anaphylaxis may occur even at substantially lower exposure levels in susceptible individuals [12,13,14,15,16,17,18,19].

**Figure 1:**
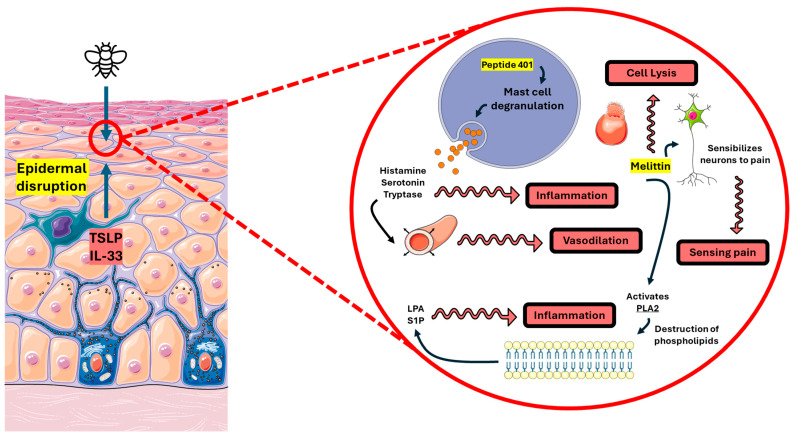
Mechanism of action of bee venom on human skin: upon injection, the venom triggers the degranulation of mast cells, releasing histamine and causing inflammation. Melittin activates the enzyme PLA2, leading to cell lysis and intensifying the inflammatory response, which manifests as symptoms such as itching, warmth, pain, and the formation of papules.

BV harbors many active compounds, including peptides and enzymes such as melittin, phospholipase A2 (PLA2), apamin, and mast cell degranulating peptide (MCDP), which exhibit remarkable potential in addressing inflammation and Central Nervous System ailments like Parkinson’s disease (PD), Alzheimer’s disease (AD), and amyotrophic lateral sclerosis [1,20,21,22]. Figure 1 shows some effects of BV, and Table 1 shows some main components of BV and their effects. In addition to these therapeutic properties, BV has demonstrated encouraging efficacy in combating various forms of cancer [23,24,25,26,27] and displaying antiviral activity, even proving effective against viruses such as the human immunodeficiency virus [28,29].

The effects of bee venom are primarily mediated by its major bioactive components, each exhibiting distinct molecular targets and mechanisms of action. Melittin, the main peptide of bee venom, is an amphipathic α-helical peptide capable of interacting directly with lipid bilayers, promoting pore formation, membrane destabilization, and increased cellular permeability [1,36]. Through these membrane interactions, melittin may access intracellular signaling pathways involved in apoptosis, oxidative stress, mitochondrial dysfunction, and inflammatory regulation, including NF-κB, MAPK, and c pathways [52,53]. Phospholipase A2 (PLA2) hydrolyzes membrane phospholipids, generating arachidonic acid and lysophospholipids that modulate inflammatory and immune responses and influence membrane remodeling and intracellular signaling cascades. Apamin, a small neurotoxic peptide, selectively blocks Ca^2+^-activated small-conductance potassium (SK) channels, thereby modulating neuronal excitability, synaptic transmission, and neuroinflammatory responses. In contrast, hyaluronidase primarily degrades extracellular matrix hyaluronic acid, increasing tissue permeability and facilitating the diffusion and bioavailability of other venom components [54,55]. Collectively, these mechanisms contribute to the pleiotropic biological activities of bee venom, although they may also underlie its cytotoxic and dose-dependent adverse effects [54].

Although venom-based therapies may entail high costs and the potential for adverse reactions due to allergies [46,47,48], the fundamental biological properties and mechanisms of action of BV make it a promising therapeutic choice for alternative treatment approaches to combat human diseases [8,37,41].

### Bee Venom and Its Possible Applications

BV consists of 88% water, and in dry venom, there are at least 18 active components, including enzymes, peptides, and biogenic amines. The predominant constituents of BV include melittin (the most abundant compound), apamin, secapin, procamine, adolapin, and MCDP [55,56,57,58]. PLA2 stands out as the main enzymatic component of BV, while other enzymes such as hyaluronidase, acid phosphomonoesterase, lysophospholipase, and α-glucosidase are present, although in smaller proportions. Furthermore, BV contains several non-peptide components that affect cellular systems, such as histamine, dopamine, and norepinephrine, as well as sugars, including fructose and glucose, and phospholipids [59,60,61].

Currently, BV is considered well-chemically characterized. However, isolated groups of their chemical compounds are studied to varying degrees, with considerable attention given to proteomics and peptide composition. In contrast, scarce information about their metabolic composition can be found in the literature [62]. In addition to peptides and enzymes, BV also contains low-molecular-weight volatile and extractive compounds, including amines, amino acids, phospholipids, and bioactive lipids. Although present in smaller quantities, these molecules may contribute to the overall pharmacological profile of bee venom by modulating inflammatory responses, oxidative stress, membrane permeability, and cellular signaling pathways. Their interactions with major venom peptides may also influence the biological and therapeutic effects described in chronic inflammatory, metabolic, and neurodegenerative disorders [1,7,18].

BV and its main constituents exhibit a wide range of pharmacological properties, including anticancer, antibacterial, anti-inflammatory, antioxidant, antiviral, radioprotective, antinociceptive, and antifungal properties, making it a promising agent in various therapies [8,39,63,64]. In the treatment of neurodegenerative diseases such as PD, AD, and amyotrophic lateral sclerosis, BV has shown neuroprotective potential by modulating inflammatory responses in the nervous system, protecting against oxidative stress, and regulating signaling pathways involved in neuronal survival [65,66,67,68,69].

Furthermore, BV has been extensively investigated for its positive effect on inflammatory and autoimmune conditions, such as rheumatoid arthritis (RA). Studies show that BV can inhibit the production of pro-inflammatory cytokines and regulate immune cell activity, contributing to the relief of symptoms associated with chronic inflammation [63,70,71]. In the dermatological field, BV has been explored for the treatment of skin diseases such as psoriasis and dermatitis due to its ability to modulate the cutaneous inflammatory response and promote tissue regeneration [72]. Additionally, BV has shown efficacy in controlling musculoskeletal pain, and it is being used as a complementary therapy to relieve pain associated with muscle and joint injuries [45,73].

**Figure 2:**
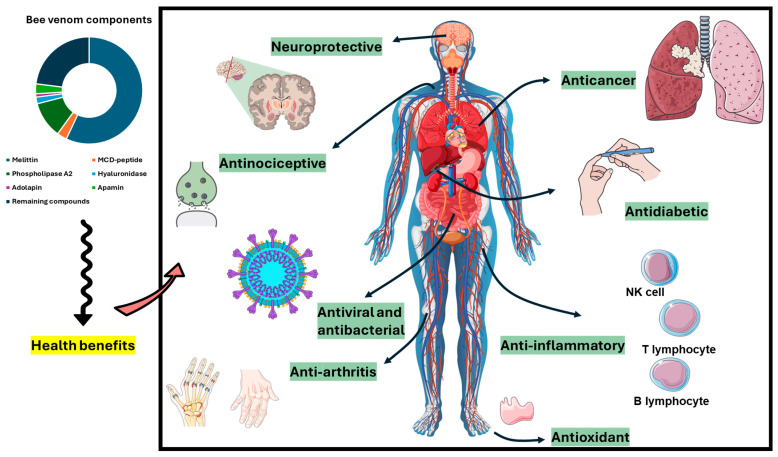
Overview of the major pharmacological activities and therapeutic research domains associated with bee venom and its bioactive components. Experimental and preclinical studies have reported anti-inflammatory, anticancer, neuroprotective, antinociceptive, antimicrobial, antioxidant, anti-arthritic, and antidiabetic properties mediated through multiple molecular and cellular pathways. The figure summarizes broad biomedical applications currently under investigation and should not be interpreted as representing established clinical indications or definitive therapeutic efficacy.

The applications of BV also extend to the oncological field, where melittin has demonstrated effectiveness in inducing apoptosis, inhibiting cell proliferation, blocking angiogenesis, and reducing metastasis [36,74] (Figure 2). In the metabolic context, BV has been studied as a potential therapy for diabetes control, showing the ability to improve insulin secretion and reduce blood glucose levels in experimental models [75,76,77].

This review presents significant differences compared to recent reviews, mainly by adopting an integrative approach that encompasses multiple chronic diseases, such as cancer, diabetes, and neurological disorders, instead of focusing on only one specific condition. Furthermore, it stands out for its strong mechanistic exploration, articulating various molecular pathways such as NF-κB, MAPK, Nrf2/HO-1, and TLR4, and for the association between specific venom compounds (melittin, apamin, PLA2) and their biological effects. Another positive point is the incorporation of translational aspects, such as nanoparticle delivery systems, and a consistent critical analysis of limitations, toxicity, and clinical gaps, something not always present in similar reviews. In summary, this review aims to summarize and discuss the current evidence regarding the therapeutic potential of BV and its major bioactive compounds in cancer, diabetes, and neurodegenerative disorders, with emphasis on their molecular mechanisms, biological activities, and translational challenges.

The literature search was conducted in the PubMed, EMBASE and Cochrane databases using combinations of the keywords “bee venom”, “melittin”, “phospholipase A2”, “apamin”, “hyaluronidase”, “cancer”, “diabetes”, “neurodegenerative diseases”, “inflammation”, “oxidative stress” and “therapeutic applications”. Original articles, experimental studies, clinical investigations, and relevant review papers published primarily in English were considered. Priority was given to studies addressing molecular mechanisms, pharmacological activities, therapeutic potential, toxicological aspects, translational limitations, and emerging therapeutic strategies involving bee venom and its major bioactive compounds. Duplicate studies, articles lacking direct relevance to the scope of the review, and reports with insufficient methodological information were excluded.

## 2. Bee Venom and Inflammation, Oxidative Stress and Diseases

### 2.1. Bee Venom and Inflammation

Inflammation is a complex and essential response of the immune system to tissue damage, infections, and external stimuli, characterized by the activation of a cascade of events that includes the release of pro-inflammatory cytokines, the recruitment of immune cells, and the activation of intracellular pathways. While this response is crucial for the body’s defense, it can become harmful if not properly controlled, leading to chronic conditions that contribute to various diseases [78,79,80,81,82]. To mitigate the adverse effects of this response, anti-inflammatory treatments, such as non-steroidal anti-inflammatory drugs (NSAIDs) and corticosteroids, are often used. However, these treatments have limitations and side effects that limit their prolonged use, such as excessive immune suppression and increased susceptibility to infections [83]. In this context, the search for alternative therapies, such as BV, has gained attention, as it contains bioactive compounds that can modulate inflammation without the severe adverse effects associated with conventional treatments [84,85,86].

Chung et al. [87] conducted a series of in vitro and in vivo tests to evaluate the effects of essential BV (eBV), a derivative of BV developed by removing PLA2 and histamine to reduce the occurrence of allergic reactions in models of inflammation. In vitro tests were performed on RBL-2H3 cells stimulated by compound 48/80 and RAW 264.7 murine macrophage cells stimulated by lipopolysaccharide (LPS) to investigate the effects of eBV on the production of pro-inflammatory cytokines in the activation of intracellular signaling, such as the NF-κB (nuclear factor kappa B) pathway and IRF3 (Interferon Regulatory Factor 3 Polyclonal Antibody). In in vivo tests, a carrageenan-induced edema model in rats was used. In this model, eBV was administered to evaluate its ability to reduce edema, inflammatory cell infiltration, and pro-inflammatory cytokine production. The results of these tests indicated that eBV has potent anti-allergic and anti-inflammatory effects, both in vitro and in vivo, mediated by modulation of the IRF3 signaling pathway. These findings suggest that eBV may represent a promising new therapy for the treatment of allergic and inflammatory conditions, as its use is expected to reduce clinical adverse effects, including pain and swelling.

In another study, the authors [60] investigated the anti-inflammatory potential of BV from the species *Apis mellifera intermissa* collected in different regions of Morocco. The in vitro tests evaluated the effects of this substrate on the pro-inflammatory response in the RAW264.7 macrophage cell line. The results showed significant anti-inflammatory activity in all venom samples tested, with notable differences between samples from the South and Northeast regions. Samples from the Northeast region demonstrated the best inhibitory activity on nitric oxide (NO) production, indicating a strong anti-inflammatory effect. On the other hand, samples from the South region showed the lowest activity, possibly due to a lower content of melittin and PLA2. Furthermore, the results suggested a negative relationship between anti-inflammatory activity and the presence of PLA2 and a moderately negative correlation with apamin, highlighting the complexity of the interactions between the different components of BV in its therapeutic properties.

Researchers evaluated the effects of BV administered at a dose of 60 mg/kg as a potential antiarthritic and anti-inflammatory therapy. They compared the efficacy of BV with that of methotrexate and investigated the mechanisms underlying its action in rats with complete Freund’s adjuvant-induced arthritis. Assessment parameters included signs of arthritis and laboratory measurements, such as erythrocyte sedimentation rate and serum levels of inflammatory cytokines, including tumor necrosis factor-α (TNF-α) and interleukin-1β (IL-1β). Furthermore, histopathological examinations and NF-κB (P65) immunostaining were performed on the affected knee joints. In vitro tests were conducted to evaluate BV’s anti-inflammatory and analgesic activity, including cyclooxygenase (COX) inhibition, carrageenan paw edema test, and acetic acid writhing tests. The results of this study highlighted the potential antiarthritic, anti-inflammatory, and antinociceptive mechanisms of action of BV for the treatment of arthritis. The findings of the studies have shown that it exerts its effects through the inhibition of key inflammatory axes, including the combined reduction in serum levels of TNF-α, IL-1β, and NF-κB, as well as inhibition of the COX-2 signaling pathway [88].

Gu et al. [89] investigated the effects of BV and melittin in models of acne vulgaris induced by insulin-like growth factor 1 (IGF-1) or *Cutibacterium acnes* (*C. acnes*). Exposure to *C. acnes* or IGF-1 induces lipogenesis, sebum production, and inflammatory reactions by activating the protein kinase B (Akt)/mammalian target of rapamycin (mTOR)/sterol regulatory element-binding protein (SREBP) signaling pathway (a pathway that controls protein synthesis through the activation and phosphorylation of lipogenic genes), both in vivo and in vitro. This study demonstrated that BV and melittin effectively suppressed inflammatory responses by regulating Akt/mTOR/SREBP signaling and suppressed lipogenesis by modulating lipogenic processes. Thus, BV and melittin emerged as potential anti-acne agents, targeting both associated inflammation and lipogenesis.

In the study by Zheng et al. [70], the researchers developed a folic acid-modified, melittin-loaded solid lipid nanoparticle (Fa-MpG@LNP) to enhance targeted delivery of melittin to activated macrophages. In vitro experiments demonstrated that Fa-MpG@LNP effectively reduced the production of pro-inflammatory cytokines, such as TNF-α, IL-1β, and IL-6, in lipopolysaccharide (LPS)-stimulated macrophages, indicating its potential to mitigate excessive inflammatory responses. In vivo evaluations in LPS-induced acute pneumonia models showed that intravenous administration of Fa-MpG@LNP significantly reduced cytokine levels in inflamed tissues and serum, suggesting a systemic anti-inflammatory effect. Furthermore, local and systemic toxicity tests revealed no detectable toxic side effects in the major organs of the mice, indicating that the developed formulation can be safely applied in the treatment of acute inflammatory reactions.

Praphawilai et al. [90] investigated the effects of melittin, derived from the venom of *Apis mellifera* and *Apis florea*, on RAW 264.7 macrophage cells stimulated by LPS. Exposure to LPS induced the production of inflammatory mediators, such as NO and pro-inflammatory cytokines, by the simulation of the mitogen-activated protein kinase (MAPK) signaling pathway. The results showed that melittin, at non-cytotoxic concentrations, significantly inhibited the expression of the inducible nitric oxide synthase (iNOS), COX-2, and IL-6 genes, demonstrating a negative regulation of the inflammatory response. Furthermore, melittin reduced NO production in a dose-dependent manner, highlighting its potential as an anti-inflammatory agent. In parallel, melittin also exhibited notable antiviral actions against herpes simplex viruses types 1 and 2 (HSV-1 and HSV-2). It inhibited viral infectivity by interfering with multiple stages of the viral replication cycle, suggesting a distinct mechanism of action from conventional nucleoside drugs.

In a recent study, Xing et al. [91] evaluated the potential neuroprotective effects of melittin in cerebral ischemia models, focusing on its anti-inflammatory mechanisms. In vivo tests in C57BL/6 mice subjected to induced ischemia showed that melittin administration significantly reduced infarct volume, improved cerebral blood flow, and reduced neurological deficits. This anti-inflammatory effect was associated with the upregulation of monocyte chemotactic protein-1 induced protein (MCPIP1), which inhibits NF-κB signaling and reduces levels of pro-inflammatory cytokines, such as IL-1β, IL-6, and TNF-α. In vitro experiments on BV-2 microglial cells confirmed these findings, demonstrating that melittin effectively suppressed NF-κB nuclear translocation and cytokine expression. These results suggest that melittin has potential as an anti-inflammatory agent, possibly capable of attenuating ischemia-induced injuries through MCPIP1-regulated pathways.

The study by Liu et al. [71] presented an innovative delivery system of melittin gel (MLT-Gel@HC-EA) for the treatment of rheumatoid arthritis in animal models, utilizing a pH-responsive gel that releases melittin in a controlled manner in an acidic environment of RA. The technique combined acupuncture with drug delivery using a hollow needle (HC needle). The results showed a significant reduction in paw and joint thickness, and less cartilage erosion and infiltration of inflammatory cells. The quantification of pro-inflammatory cytokines, such as TNF-α and IL-1β, indicated that the treatment inhibited these molecules, suggesting an anti-inflammatory effect. Furthermore, MLT-Gel@HC-EA restored immune balance by reducing Th17 cell activation and increasing the proportions of regulatory T cells (Treg) and M2 macrophages. The analysis of signaling pathways revealed that MLT-Gel@HC-EA modulated the NF-κB pathway, contributing to the regulation of immune responses.

Taken together, these studies suggest that bee venom and melittin may exert broad anti-inflammatory effects through convergent mechanisms, particularly the downregulation of NF-κB, MAPK, COX-2, iNOS, and pro-inflammatory cytokines. The section is strengthened by the inclusion of both cellular and animal evidence across distinct inflammatory conditions, which supports biological plausibility. However, the evidence remains largely preclinical, limiting direct clinical translation. Considerable heterogeneity is also evident in venom source, composition, formulation, dose, route of administration, and experimental models, making comparisons difficult and preventing firm conclusions about efficacy or safety. In addition, although some studies attempted to reduce toxicity or improve targeted delivery, the known cytotoxic and allergenic risks of bee venom remain an important concern. Therefore, while the findings are promising, the current literature still lacks standardization, robust pharmacological characterization, and well-designed clinical trials to establish therapeutic relevance in humans.

### 2.2. Bee Venom and Oxidative Stress

Oxidative stress is a critical factor in various health conditions, characterized by an imbalance between the production of reactive oxygen species (ROS) and the body’s ability to neutralize them. This phenomenon is associated with inflammatory and apoptotic processes that can lead to cellular degeneration [86,92,93,94]. Currently, conventional treatments for these conditions often focus on alleviating symptoms but do not directly address the underlying cause of oxidative stress. In this scenario, BV emerges as a promising alternative due to its antioxidant, anti-inflammatory, and neuroprotective properties, attributed to components such as melittin, PLA2, and apamin [41,95,96,97,98].

Some authors explored the effects of BV on HT22 cells exposed to oxidative stress induced by amyloid beta peptide 1–42 (Aβ1–42). The researchers focused on two cellular signaling pathways: the Nrf2/HO-1 (factor erythroid 2-like 2/heme oxygenase-1) pathway and the TrkB/CREB/BDNF (tropomyosin-related kinase receptor B/cAMP response element-binding/brain-derived neurotrophic factor) pathway, related to neuronal growth and survival. They conducted experiments using laboratory-grown neuronal cells and exposed them to Aβ1–42 to induce oxidative stress. They then treated these cells with BV to evaluate its protective effects. The results showed that BV activated both the Nrf2/HO-1 pathway and the TrkB/CREB/BDNF pathway in neuronal cells exposed to oxidative stress. This was evidenced by the increased expression of proteins associated with these pathways, such as heme oxygenase-1 (HO-1) in the Nrf2/HO-1 pathway and brain-derived neurotrophic factor (BDNF) in the TrkB/CREB/BDNF pathway. Furthermore, it was observed that treatment with BV reduced oxidative stress in neuronal cells, demonstrating its antioxidant capacity. This reduction in oxidative stress has also been associated with protection against cellular damage induced by Aβ1–42. These results suggest that BV has neuroprotective properties against oxidative stress induced by Aβ1–42, mediated by the activation of the Nrf2/HO-1 and TrkB/CREB/BDNF pathways in neuronal cells. Therefore, BV may be a potential therapeutic strategy for treating neurodegenerative disorders [99].

In another study, they sought to investigate the effects of melittin on HT22 neuronal cells in culture and in an animal model of cognitive deficits induced by Aβ 25–35, a protein associated with AD. The results revealed that melittin could protect HT22 cells against oxidative stress caused by Aβ 25–35, reducing the production of ROS within the cells and preventing cell apoptosis. Through molecular analyses, melittin was found to positively regulate the expression of pro-apoptotic factors and promote the activation of the antioxidant pathway, including the nuclear translocation of the transcription factor Nrf2 and the subsequent expression of the antioxidant enzyme HO-1. Furthermore, melittin has been shown to positively influence neuronal activity by activating the TrkB/CREB/BDNF pathway, which contributes to neurogenesis and regulates synaptic function. These effects were corroborated by experiments in mice, in which it significantly improved learning and memory capacity, especially in animals with cognitive deficits induced by Aβ 25–35. Melittin also increased neurogenesis in the hippocampus. Additional analyses in mouse brain tissue and serum confirmed the beneficial effects of melittin, including restoration of the balance between oxidants and antioxidants, the activity of the cholinergic system, and the regulation of intercellular neurotrophic factors. These results suggest that melittin has therapeutic potential as a neuroprotective agent against neurodegenerative diseases [100].

Kim et al. [101] were the first to study the antioxidant, antiapoptotic, and anti-inflammatory effects of apamin, one of the constituents of BV, on acute kidney injury (AKI) induced by LPS in male C57BL/6N mice. Apamin promoted significant improvements in renal dysfunction and histological injury, especially tubular injury, in mice injected with LPS. It also suppressed oxidative stress induced by LPS through modulating the expression of nicotinamide adenine dinucleotide phosphate oxidase 4 (NOX4) and HO-1. Tubular cell apoptosis, characterized by caspase-3 activation in LPS-injected mice, was significantly attenuated by apamin. Additionally, it was observed that apamin inhibited the production of pro-inflammatory cytokines (TNF-α, IL-6) and the accumulation of immune cells, also suppressed the toll-like receptor 4 (TLR4) pathway, and significantly decreased levels of vascular cell adhesion molecule-1 (VCAM-1) and intercellular adhesion molecule-1 (ICAM-1).

The potential of BV to combat oxidative stress induced by zinc oxide nanoparticles (ZNPs) in the brain of rats was evaluated in one study [102]. The results indicated that BV significantly reduced depression, anxiety, memory impairment, and spatial learning disorders induced by ZNPs. The increase in serotonin and dopamine levels and Zn content induced by ZNPs was significantly suppressed. BV significantly restored depleted total antioxidant capacity (TAC) and minimized the increase in brain malondialdehyde (MDA) associated with exposure to ZNPs. Furthermore, the neurodegenerative changes induced by ZNPs were significantly reduced by BV, which also alleviated the increase in neurofilament and GAP-43 protein immunoexpression due to exposure to ZNPs. These results suggest that BV may be a biologically effective therapy to mitigate the neurotoxic and neurobehavioral effects of ZNPs, mainly when administered during exposure to ZNPs.

Badr et al. [103] analyzed the effects of BV on wound healing in diabetic mice, focusing on oxidative stress pathways mediated by the transcription factors ATF-3 and iNOS. Diabetic mice exhibited increased expression of ATF-3 and iNOS in injured tissues, associated with elevated levels of free radicals and compromised healing. BV treatment resulted in significant downregulation of these markers, reducing oxidative stress and restoring balance in ROS levels. As a result, BV accelerated wound closure and enhanced neovascularization by increasing endothelial progenitor cell recruitment, revealing its antioxidative properties as a promising intervention in diabetic wound management.

In the study by Lomeli-Lepe et al. [9], researchers investigated the effects of BV in a lipopolysaccharide-induced animal model of synucleinopathies, focusing on inflammatory responses and oxidative stress in the substantia nigra (SN) and striatum (STR) regions. The results indicated that BV significantly reduced α-syn expression and increased TH levels, a key enzyme in dopamine synthesis, suggesting a possible restoration of neuronal function. Additionally, a decrease in microglial and astroglial activation was observed, along with reduced IL-1β levels and lipid peroxidation, which was associated with increased catalase activity.

Zan et al. [104] investigated the protective role of melittin in sepsis-induced AKI, focusing on its ability to inhibit ferroptosis, a type of regulated cell death associated with lipid peroxidation. In LPS-induced AKI models, treatment with melittin resulted in significant reductions in serum markers of renal dysfunction, such as creatinine and blood urea nitrogen, and alleviated histological signs of tubular injury, including tubular dilation and cellular swelling. Melittin promoted the expression of Glutathione Peroxidase 4 (GPX4), a crucial antioxidant enzyme that protects cells against oxidative stress, particularly in lipid peroxidation, by enhancing the nuclear translocation of the transcription factor NRF2. This NRF2 activation was associated with the positive regulation of antioxidant genes, including GPX4, which plays a central role in ferroptosis resistance. The results showed reduced levels of lipid ROS and MDA, as well as decreased iron accumulation in renal tissues, indicating a reduction in oxidative stress.

In a recent study, Mao et al. [98] investigated the therapeutic effects of melittin on oxidative stress in Schwann cells (SCs) subjected to hypoxic conditions. The research established a model of oxidative stress using CoCl_2_ to induce injury in SCs, demonstrating that melittin significantly mitigated oxidative damage by downregulating the expression of inflammatory cytokines, particularly IL-1β and TNF-α. This suppression was associated with the inhibition of the NF-κB signaling pathway. Furthermore, treatment with melittin resulted in a significant increase in the expression of hypoxia-inducible factor 1-alpha (HIF-1α), suggesting a potential protective mechanism against hypoxia-induced injuries.

Collectively, these findings indicate that bee venom and its major peptides may attenuate oxidative stress through multiple complementary pathways, including activation of Nrf2/HO-1, upregulation of GPX4, reduction in lipid peroxidation, and modulation of apoptosis- and inflammation-related signaling. The section is strengthened by the inclusion of diverse experimental models of neurodegeneration, kidney injury, hypoxia, wound healing, and nanoparticle-induced toxicity, thereby broadening the mechanistic relevance of the evidence. However, the overall body of literature remains predominantly preclinical and highly heterogeneous regarding models, venom fractions, dosages, and delivery methods. Some mechanistic descriptions also overlap with anti-inflammatory effects, making it difficult to isolate antioxidant-specific actions. In addition, improvements in biochemical markers do not necessarily guarantee clinically meaningful outcomes in humans. Thus, despite promising antioxidant and cytoprotective signals, the translational value of bee venom remains uncertain until standardized formulations, clearer dose–response data, and rigorously designed clinical studies confirm efficacy and safety.

### 2.3. Bee Venom and Cancer

Cancer diseases represent one of the major challenges to global public health, with high rates of incidence and mortality. The pathophysiology of cancer is complex, involving the dysregulation of normal cellular processes such as proliferation, apoptosis, and cell migration [16,105,106,107,108,109,110]. Conventional treatment, which includes chemotherapy, radiotherapy, and surgery, often has limitations, such as treatment resistance and significant side effects. In this landscape, the search for complementary and alternative therapies has gained prominence, particularly regarding the use of natural compounds. Bee venom, in particular, has received attention for its ability to induce apoptosis, inhibit cell proliferation, and modulate signaling pathways related to cancer [17,23,37,111,112].

The cytotoxic effect of BV in combination with cisplatin on the 4T1 invasive mammary carcinoma cell line was evaluated by Shiassi Arani et al. [113]. For this analysis, cells were treated with various concentrations of BV or cisplatin alone and in combination (BV/cisplatin). The results revealed that combining these two agents produced a remarkable synergistic effect, reducing cancer cell viability and inducing programmed cell death (apoptosis). This suggests that BV may increase the effectiveness of cisplatin in treating breast cancer. Additionally, the study noted that BV exhibited antimutagenic effects, suggesting it may help prevent or reduce genetic mutations associated with cancer development.

The effects of BV have also been evaluated in pancreatic cancer. The results obtained showed that BV exerted a notable suppression on the proliferation of pancreatic cancer cells, inducing cell cycle arrest in the S phase. Furthermore, apoptosis and cell migration were evaluated, revealing that BV treatment significantly promoted apoptosis and inhibited pancreatic cancer cell migration. This study also demonstrated that BV effectively inhibited the proliferation of pancreatic cancer cell lines AsPC-1 and PANC-1, further highlighting its therapeutic potential. Furthermore, flow cytometric analysis revealed cell cycle changes induced by BV, suggesting an association between inhibition of cell proliferation and cycle regulatory proteins. An increase in the protein expression of p53 and p21 was also observed, indicating their antitumor effect on pancreatic cancer cells. There was a reduction in the expression of Bcl-2 and an increase in the expression of Bax, in addition to the rise in the expression of cleaved caspase-3 and cleaved caspase-9, suggesting the activation of apoptotic pathways [114].

Apamin, one of the constituents of BV, has also demonstrated significant anticancer potential, as evidenced by the study conducted by Badr-Eldin et al. [115]. In this work, the authors explored apamin-functionalized emulsomes (EGA-EML-APA) with the aim of enhancing the cytotoxicity of ellagic acid in MCF-7 breast cancer cells. The results obtained revealed that EGA-EML-APA exhibited an IC50 of 5.472 ± 0.21 μg/mL, which is considerably superior to the value of 9.09 ± 0.34 μg/mL observed for free ellagic acid. Chemically, apamin appears to facilitate the cellular penetration of ellagic acid, thereby enhancing its efficacy. Furthermore, the formulation demonstrated the ability to induce apoptosis, as evidenced by the increased expression of markers such as p53, bax, and caspase-3, while the synthesis of the anti-apoptotic protein bcl-2 was reduced. The inhibition of NF-κB activity, along with the increase in TNF-α, reinforces the apoptotic activity of EGA-EML-APA.

In an investigation conducted by Chahla et al. [77], the anticancer potential of *Apis mellifera syriaca* venom was explored using both in vitro and in vivo glioblastoma models. In the in vitro assays, the venom demonstrated strong cytotoxic activity against U87 glioblastoma cells, with IC50 values of 14.32 μg/mL in the 3-(4,5-dimethylthiazol-2-yl)-2,5-diphenyl tetrazolium bromide (MTT) assay and 7.49 μg/mL in the lactate dehydrogenase (LDH) assay, indicating nearly complete inhibition of cell viability at concentrations above 25 μg/mL. The observed mechanisms of cell death included the induction of cellular stress and compromise of membrane integrity, leading to a pattern of cell death resembling necrosis without signs of early apoptosis, as evidenced by the cleavage of poly ADP-ribose polymerase (PARP). In contrast, in vivo studies showed that venom administration in mouse models resulted in a significant reduction in tumor size, corroborating the in vitro findings and revealing an increase in caspase-3 expression, suggesting activation of apoptotic pathways. These similarities in results indicate that BV is effective in controlled laboratory settings and has a promising therapeutic impact in a more complex biological context, reinforcing its potential as a treatment for glioblastoma.

Researchers evaluated the impact of BV on non-small cell lung cancer (NSCLC) and found that BV not only inhibits the proliferation, migration, and invasion of these cells but also enhances the effect of the epidermal growth factor receptor (EGFR) tyrosine kinase inhibitor, gefitinib. Bioinformatics analysis and protein docking modeling suggested that melittin, one of the main components of BV, directly interacts with EGFR, modulating the autophagy mediated by this receptor. The results showed that BV, alone or in combination with gefitinib, significantly reduced the expression of the phosphorylated form of EGFR and increased the LC3-II/LC3-I ratio, indicating increased autophagy in NSCLC cells. These findings suggest that BV may act as a promising therapeutic agent due to its intrinsic antitumor properties and its ability to enhance the efficacy of conventional treatments, such as gefitinib [2].

A study on bee venom nanoparticles (BVNPs) derived from *Apis mellifera* venom demonstrated potential for cancer therapy, particularly against the MCF-7 breast cancer cell line. Using a hydrothermal synthesis method, the BVNPs were characterized using advanced techniques, including UV–Vis spectroscopy and transmission electron microscopy. The results demonstrated a dose-dependent decrease in cell viability, highlighting the antiproliferative activity of the BVNPs. Furthermore, treatment with these nanoparticles induced morphological characteristics of apoptosis in MCF-7 cells, such as cell shrinkage and nuclear fragmentation [3].

Sivri et al. [64] investigated the cytotoxic effects of BV and melittin on MDA-MB-231 breast cancer cells. Using MTT and scratch assays, the study demonstrated that BV and melittin exhibit selective cytotoxicity, surpassing cisplatin. Notably, the expression profiling of metastasis-related genes revealed that melittin significantly increased the expression of the anti-metastatic genes BRMS1 and DRG1, while bee venom enhanced the expression of DRG1 and KAI1/CD82. On the other hand, the prometastatic gene WNT7B was downregulated in BV-treated cells. It is concluded that the anti-metastatic effects of bee venom and melittin are primarily mediated through the upregulation of BRMS1, DRG1, and KAI1/CD82, along with the downregulation of WNT7B.

In a recent study, Badivi et al. [116] demonstrated that the formulation of BV encapsulated in polyethylene glycol-modified liposomes (BV-Lipo-PEG) exhibits an effective mechanism of action in the treatment of A549 lung cancer cells. BV-Lipo-PEG not only improved the stability and bioavailability of venom but also resulted in a significantly higher apoptotic rate in A549 cells compared to other samples. Molecular analysis revealed that, in A549 cells treated with BV-Lipo-PEG, there was a decrease in the expression levels of matrix metalloproteinase 2 and 9 (MMP-2 and MMP-9) genes, which are linked to cell proliferation and invasion, as well as the Cyclin E protein, which regulates the cell cycle. Conversely, the expression levels of caspases 3 and 9, which are crucial for apoptosis induction, increased. This targeted release and modulation of gene expression result in a higher concentration of BV in cancer cells, promoting programmed cell death and inhibiting cell proliferation, especially in the S phase of the cell cycle.

Finally, these studies indicate that bee venom and its constituents may exert relevant antitumor effects through multiple mechanisms, including inhibition of proliferation and migration, induction of apoptosis, modulation of autophagy, and enhancement of sensitivity to conventional agents such as cisplatin and gefitinib. The section is strengthened by the inclusion of several tumor models and by the consistent reporting of molecular changes involving p53, Bax/Bcl-2, caspases, EGFR, and metastasis-related genes. However, the evidence remains largely limited to in vitro experiments and animal models, which restricts direct clinical interpretation. Considerable heterogeneity in venom source, composition, formulation, dose, and cancer type also makes cross-study comparison difficult. In addition, strong cytotoxicity against tumor cells does not by itself establish selectivity, systemic safety, or therapeutic feasibility in humans. Therefore, although bee venom shows promising anticancer activity, robust translational studies and carefully designed clinical trials are still needed before any therapeutic recommendation can be justified.

### 2.4. Bee Venom and Diabetes

Diabetes mellitus is a multi-complex metabolic condition characterized by chronic hyperglycemia due to failures in insulin secretion, insulin action, or both. This condition is associated with various complications, including cardiovascular diseases, neuropathies, and endothelial dysfunctions [117,118,119,120]. Conventional treatment typically involves medications such as metformin, which reduces glucose production by the liver and improves insulin sensitivity. As demonstrated in recent studies, BV has emerged as a promising alternative, exhibiting properties that may modulate inflammatory processes and protect against oxidative damage [121,122,123,124,125].

Some researchers have compared the effects of metformin and BV in diabetic mice. The results demonstrated a significant increase in glucose levels and a significant decrease in insulin levels in diabetic mice, reversed by treatment with metformin and BV. The elevated TNFα, IL6, and IL10 levels observed in the diabetic mice were also significantly reduced with metformin and BV. Histological analysis of the pancreas revealed significant changes in the islets of Langerhans in diabetic mice, most notably restored by BV treatment [121].

Zahran et al. [126] investigated the effects of BV on cardiac dysfunction in hyperlipidemic diabetic rats, comparing its efficacy with the combined therapy of metformin and atorvastatin. The administration of BV resulted in a significant reduction in glucose, total cholesterol, and triglyceride levels in hyperlipidemic diabetic rats. Cardiac damage markers, such as troponin I and creatine kinase (CK-MB), also decreased, indicating cardiac protection. Tissue analysis revealed a reduction in MDA levels, a marker of oxidative stress, and in the expression of inflammatory mediators such as NF-κB and VCAM-1.

In another study, Al-Shaeli et al. [123] evaluated the effects of BV in mice with type 2 diabetes mellitus induced by alloxan and glucose fluids. The findings showed that HBV administration led to a significant increase in insulin and HDL levels and a reduction in glucose, cholesterol, and triglyceride concentrations. This indicates that BV may act in the modulation of lipid and glucose metabolism, contributing to improving metabolic status in diabetic models. However, the study also noted limitations, including the lack of detailed histological sections of the pancreas and liver, which hinder a more comprehensive understanding of the histopathological effects of the venom.

Radwan et al. [76] investigated the therapeutic potential of BV and bone marrow mesenchymal stem cells (BMSCs) in rats with type I diabetes mellitus. Treatment with BV demonstrated beneficial effects on the morphology of the lingual mucosa, contributing to the restoration of taste buds and the reduction in epithelial atrophy. While BV played a positive role in tissue regeneration, the most significant effects on growth factor expression, such as TGF-β1 and VEGF, were primarily attributed to BMSCs. This combination of therapies suggests promising potential for treating complications associated with diabetes, as preserving taste function may enhance patients’ quality of life and influence glycemic control through changes in eating habits.

To provide a theoretical basis for the use of melittin in the treatment of diabetic peripheral neuropathy (DPN), the study by Zhang et al. [56] investigated the effects of this substance on Schwann cells (SCs) under hyperglycemic conditions. The results showed that melittin increased cell viability and activated the Crabp2/Wnt/β-catenin signaling pathway, which is essential for cell survival. Proteomic analysis revealed 1784 proteins with altered expression, of which 725 were upregulated. Additionally, flow cytometry indicated an increase in the G2/M and S phases of the cell cycle and a decrease in the apoptosis rate, suggesting that melittin stimulates cell proliferation and inhibits programmed cell death. Studies using polymerase chain reaction (PCR) and immunofluorescence confirmed the increase in proliferation markers, such as CDK4 (cyclin-dependent kinase 4) and CyclinD1.

In addition to its anti-inflammatory and metabolic effects, emerging evidence suggests that BV may modulate intracellular signaling pathways directly involved in insulin sensitivity and glucose utilization. Experimental studies indicate that melittin and other BV-derived peptides can influence pathways associated with AMP-activated protein kinase (AMPK), phosphatidylinositol-3-kinase/protein kinase B (PI3K/Akt), and glucose transporter type 4 (GLUT4) translocation, which are critical for maintaining glucose homeostasis and peripheral insulin responsiveness. Furthermore, BV’s attenuation of oxidative stress appears to improve mitochondrial function and reduce cellular damage in metabolically active tissues, such as skeletal muscle, liver, and pancreatic β-cells. These findings reinforce the hypothesis that BV may act through interconnected metabolic and inflammatory pathways involved in diabetes progression and insulin resistance [19,127,128].

Another important aspect is the role of BV in regulating oxidative stress, a major contributor to diabetes-associated tissue injury. Chronic hyperglycemia promotes excessive production of reactive oxygen species (ROS), leading to lipid peroxidation, mitochondrial dysfunction, endothelial damage, and activation of inflammatory cascades. Several studies demonstrated that BV administration reduced oxidative stress markers, including malondialdehyde (MDA), while enhancing endogenous antioxidant defenses such as superoxide dismutase (SOD), catalase, and glutathione-related pathways [100,129,130,131]. These antioxidant effects may contribute not only to improved metabolic control but also to protection against long-term diabetic complications involving cardiovascular, neural, and renal tissues.

Recent investigations have also explored the potential synergistic effects between BV and conventional therapeutic approaches. Combined administration of BV with antidiabetic drugs or regenerative therapies, such as mesenchymal stem cells, has shown promising results in experimental models by enhancing tissue repair, modulating inflammatory responses, and improving functional outcomes [73]. In this context, BV-derived compounds may eventually serve as adjunctive therapies capable of potentiating existing treatments while targeting inflammatory and oxidative mechanisms that are not fully controlled by conventional glucose-lowering drugs alone. However, the safety profile of these combinations remains insufficiently characterized, particularly regarding immunogenicity, systemic toxicity, and long-term administration.

Despite the encouraging preclinical evidence, important translational limitations remain. Most available studies rely on chemically induced animal models of diabetes, which do not fully reproduce the complexity and heterogeneity of human diabetes mellitus. In addition, substantial variability exists regarding venom composition, purification methods, dosage regimens, treatment duration, and administration routes, limiting reproducibility across studies. The majority of investigations also involve short-term interventions, with limited assessment of chronic toxicity, pharmacokinetics, or immune-related adverse effects. Therefore, future studies should prioritize standardized experimental protocols, mechanistic validation, and carefully designed clinical trials to determine whether BV or its isolated components can be safely integrated into therapeutic strategies for diabetes and its associated complications.

In summary, current findings indicate that bee venom and melittin may confer favorable metabolic and tissue-protective effects in experimental models of diabetes, including improved glycemic control, better lipid parameters, reduced inflammation, and attenuation of diabetes-associated complications. A strength of this section is that it covers evidence related not only to whole-body metabolic regulation but also to specific diabetic complications, including cardiac damage, neuropathy, and injury to oral tissues. Even so, the evidence base remains constrained by the predominance of animal and in vitro studies, limiting direct translation into clinical practice. Substantial variability across diabetic models, bee venom formulations, comparator groups, and assessed outcomes also makes it difficult to identify which effects are consistent and clinically meaningful. In several reports, the mechanistic data are encouraging but still early-stage, while key aspects such as histopathological confirmation, dose consistency, and long-term safety have not been adequately investigated. Therefore, although bee venom shows potential as an adjunctive strategy in diabetes research, more robust translational and human evidence is needed before firm therapeutic implications can be established.

### 2.5. Bee Venom and Neurological Diseases

The increasing incidence of neurological diseases, including neurodegenerative disorders, represents a growing problem, affecting millions of people worldwide and being the second main cause of death and the main cause of disability, according to the Global Burden of Disease (GBD) [132]. These conditions impose a significant economic burden and present complex challenges in diagnosis and treatment, which often focus on symptom relief without addressing the underlying causes [79,133,134,135]. In this regard, BV stands out for its anti-inflammatory and neuroprotective properties, as recent studies have shown that its components may offer new perspectives in the treatment of neurological diseases [69,124,136,137].

The impact of BV phospholipase A2 (bvPLA2) on mitigating memory deficits in Tg2576 mice was investigated. Animals received bvPLA2 in concentrations ranging from 1mg/kg. The results demonstrated that bvPLA2 not only ameliorated the memory impairments observed in the mice but also led to significant reductions in Aβ, amyloid precursor protein (APP), β-secretase 1 (BACE1), and β-secretase activity. Additionally, bvPLA2 reduced pro-inflammatory cytokines and inflammation-related proteins (including p-STAT3) expression in the treatment mice, whereas anti-inflammatory cytokines and mediators’ expression increased. Finally, in cultured BV-2 cells, bvPLA2 effectively restrains Aβ production. Molecularly, findings from docking experiments, pull-down assays, and luciferase assays collectively indicated that bvPLA2 directly bound STAT3 (Signal transducer and activator of transcription-3), thereby modulating gene expression levels. Furthermore, the concurrent administration of the STAT3 inhibitor and bvPLA2 demonstrates heightened synergistic effects on both anti-amyloidogenic and anti-inflammatory outcomes, surpassing the results seen with individual administrations [138].

A study reported that bvPLA2, an inducer of Foxp3+ regulatory T-cells, offered neuroprotection at various concentrations to dopaminergic neurons by modulating neuroinflammatory responses in the 1-methyl-4-phenyl-1,2,3,6-tetrahydropyridine mouse model of PD. This neuroprotective effect of bvPLA2 has been linked to the deactivation of microglia and a reduction in CD4(+) T-cell infiltration. The findings demonstrated a direct binding between bvPLA2 and CD206 on dendritic cells, leading to the subsequent secretion of PGE2 (Prostaglandin E2). This process resulted in regulatory T-cell differentiation through the PGE2 (EP2) receptor signaling in Foxp3(−)CD4(+) T cells. These insightful observations indicate that bvPLA2-CD206-PGE2-EP2 signaling is crucial for fostering immune tolerance by promoting regulatory T-cell differentiation. Consequently, this mechanism significantly safeguards against diverse neurodegenerative disorders, including PD [139].

In another investigation, the researcher studied the effects of BV in a mouse model of LPS-induced neuroinflammation and memory loss, and BV-2 microglial cells and astrocytes. The results demonstrated that BV reduced memory impairments in the treated animals, ameliorating mouse performance in behavioral tests and preventing neuronal cell death. Additionally, BV curbed the LPS-induced rise in Aβ levels, β- and γ-secretase activities, and NF-κB DNA-binding activity in the mice’s brains. Furthermore, BV hindered the overexpression of the neuroinflammatory proteins COX-2, iNOS, glial fibrillary acidic protein (GFAP), and ionized calcium-binding adapter molecule 1 (IBA-1) in brain tissues and cultured cells. Molecularly, insights from pull-down assays and molecular modeling indicated that BV interacts with NF-κB [140].

Yao et al. [141] investigated the neuroprotective effects of melittin in a rat model of cerebral ischemia using middle cerebral artery occlusion (MCAO) to induce stroke. Rats were divided into control, saline, and melittin groups, receiving intraperitoneal injections of melittin before MCAO. Results showed that melittin treatment significantly increased neuron density in the cerebral cortex and reduced infarct size compared to the saline group. Treated rats also performed better in locomotor and cognitive tests. Immunohistochemical analysis revealed that melittin inhibited microglial activation and reduced pro-inflammatory cytokines, including TNF-α, IL-6, and IL-1β. Additionally, TLR4, MyD88, and NF-κB p65 levels decreased in the ischemic cortex. Melittin treatment also significantly reduced neuronal apoptosis induced by stroke.

In an investigation into the therapeutic potential of BV for PD, Hartmann et al. [142] conducted a prospective, randomized, double-blind study involving patients at Hoehn & Yahr stages 1.5 to 3. The aim was to assess the symptomatic and disease-modifying effects of monthly BV injections compared to placebo. The evolution of scores on the Unified Parkinson’s Disease Rating Scale (UPDRS III) was analyzed throughout the study period to monitor disease progression. After 11 months of monthly administration, the results indicated that BV did not significantly reduce UPDRS III scores. Furthermore, no significant differences in UPDRS-III scores were observed between the treated and placebo groups over the study period. These findings suggest that a higher frequency of administration and possibly higher individual doses of BV may be necessary to adequately explore its efficacy in treating PD, highlighting the need for future investigations with different treatment protocols.

Cho et al. [65] conducted a randomized clinical trial that explored the efficacy of acupuncture and bee venom acupuncture (BVA) as complementary treatments for idiopathic Parkinson’s disease (IPD). The results showed significant improvements in symptoms, especially in the BVA group, which demonstrated notable advancements in the UPDRS, the Berg Balance Scale, and the 30-m walking time. The acupuncture group also showed improvements on the UPDRS and the Beck Depression Inventory, whereas the control group did not show significant changes. The authors highlight that these findings corroborate previous research suggesting the benefits of these therapies and indicate that BVA may exert neuroprotective effects by modulating neurogenic inflammation. However, they acknowledge limitations, such as the small sample size and the relatively short treatment duration, which may limit the generalizability of the results.

In another clinical trial conducted by Cho et al. [143], the efficacy of combined treatment with acupuncture and BVA as an adjunct therapy for patients with IPD was analyzed. The results showed that after treatment, the active treatment group exhibited significant improvements in UPDRS parts II and III scores, as well as enhancements in patients’ quality of life, as measured by the Parkinson’s Disease Quality of Life Questionnaire (PDQL). Specifically, the active group demonstrated an average reduction of 5.2 points in the total UPDRS score compared with the conventional treatment group, which showed no significant change. Furthermore, the beneficial effects were maintained in follow-up assessments conducted 4 and 8 weeks after the end of the treatment, suggesting a prolonged duration of therapeutic effects.

The efficacy of bee venom therapy in patients with multiple sclerosis (MS) was investigated in a study conducted by Wesselius et al. [144], which utilized a randomized crossover clinical trial design. The research included 26 patients with relapsing forms of MS, who underwent 24 weeks of bee sting therapy followed by 24 weeks without treatment, or vice versa. The results showed that there was no significant reduction in the cumulative number of new gadolinium-enhancing lesions on magnetic resonance imaging (MRI) during the bee venom treatment, and the lesion load on T2* imaging continued to progress. Additionally, no improvements were observed in relapse rates, disability, fatigue, or quality of life measures among the patients. Although the therapy was well tolerated, with no serious adverse events, the findings indicate that bee venom does not provide significant clinical benefits in modulating disease activity in patients with MS.

Doo et al. [145] evaluated the efficacy and safety of combining acupuncture and BVA as an adjunctive treatment for IPD in a prospective, open-label, controlled trial. Participants who were on a stable dose of antiparkinsonian medication received treatment sessions twice a week for 12 weeks. The results showed significant improvements in the combined scores of parts II and III of the UPDRS, indicating a reduction in both motor and non-motor symptoms. Additionally, acupuncture and BVA appeared to enhance synaptic dopamine availability, aligning with previous findings and helping explain the observed functional improvements. Although postural stability, assessed using the Balance Master system, did not show significant changes, the study suggests that integrating BVA with acupuncture could provide a synergistic effect, amplify the benefits of standard treatment, and mitigate the side effects associated with medication.

Taken together, this section indicates that bee venom and its active constituents, especially bvPLA2 and melittin, may provide neuroprotective benefits by acting through anti-inflammatory, anti-apoptotic, and immunoregulatory mechanisms. These effects appear to involve pathways such as NF-κB, STAT3, TLR4/MyD88, and regulatory T-cell activity. An important strength of this body of evidence is that it includes both mechanistic animal research and clinical studies involving conditions such as Parkinson’s disease and multiple sclerosis. Nevertheless, the findings are not fully consistent. Experimental studies in animals have reported favorable effects on neuroinflammation, amyloid accumulation, neuronal preservation, and behavioral performance, whereas clinical studies have yielded variable results, with some reporting symptom improvement and others failing to detect a clear clinical benefit. Interpretation is also complicated by substantial heterogeneity across studies, including differences in the type of intervention, dosage, frequency of administration, and overall study design, particularly in protocols combining bee venom with acupuncture. Thus, although the mechanistic foundation is promising, the current evidence in humans remains too limited to draw definitive therapeutic conclusions, and more robust, well-designed clinical trials are required to determine efficacy, safety, and the most appropriate treatment regimens.

## 3. Bee Venom in Clinical Trials

BV has been the subject of human investigation. Some authors have evaluated the effects of BV phonophoresis applied to surgical incisions and specific acupuncture points on pain, inflammation, and hip mobility after inguinal hernioplasty. Patients were randomly assigned to a BV phonophoresis group, which received low-intensity pulsed ultrasound with BV gel, and a control group. The results showed highly significant differences in the Visual Analog Scale (VAS), C-reactive protein (CRP), and range of motion (ROM) measurements of the hip between the BV phonophoresis group and the control group. This study design not only allowed for a rigorous evaluation of BV’s efficacy in alleviating pain and inflammation but also highlighted its potential as a non-invasive therapeutic alternative in post-operative contexts [146].

A study conducted by Lee et al. [147] was the first randomized clinical trial to investigate the feasibility of combined therapy with BV and NSAIDs, specifically loxoprofen, in patients with non-specific chronic neck pain (NCNP). After 3 weeks of treatment, all groups (BV, NSAIDs, and combination) showed significant improvements in pain intensity and functional disability. However, by the end of the fourth week, the group that received BV alone demonstrated the greatest reduction in pain intensity and disability index compared to the NSAIDs and combination groups. Furthermore, the combined group continued to show a decrease in discomfort in the eighth week of follow-up, whereas the group that received only loxoprofen showed a more limited effect after treatment ended. Improvements in depressive mood and quality of life were observed in all groups, with the BV group showing the best results. These observations suggest that BV therapy may offer significant and lasting benefits for patients with NCNP.

Researchers also evaluated the efficacy and safety of purified BV (Apis mellifera) dermal injections in patients with knee osteoarthritis. After a dose escalation period, participants received 12 weekly injections, each containing 100 mg of BV. The results showed an average reduction of 3.2 points on the WOMAC (Western Ontario and McMaster Universities Osteoarthritis Index) pain scale and an improvement of 2.8 points in physical function compared to the control group that received histamine. Although 22.4% of patients in the venom group reported adverse events, most were mild and related to the injection site, indicating an acceptable safety profile. The findings suggest that BV has anti-inflammatory properties in addition to pain relief, providing significant benefits for patients who do not respond to conventional treatments [148].

The efficacy of BV as an adjunct in the treatment of chronic low back pain was evaluated in a randomized clinical trial that investigated its use in combination with NSAIDs. Participants were randomly allocated into the group that received BV injections, while the other group received sham treatment with saline solution, both administered at acupuncture points over three weeks. The efficacy of the treatment was measured using standardized assessments, including the VAS for pain intensity, the Oswestry Disability Index (ODI) for functional assessment, and quality of life questionnaires. The results showed that the BV-treated group significantly improved pain intensity, functionality, and quality of life compared to the placebo group [149].

In a randomized, double-blind, placebo-controlled study, the efficacy of sublingual immunotherapy (SLIT) with BV was evaluated in patients with large local reactions (LLRs) who were monosensitized to bees. Patients were randomized to receive either SLIT or a placebo for six months. The results showed that the diameter of LLRs decreased by more than 50% in 57% of patients treated with SLIT, demonstrating the effectiveness of this approach in reducing allergic reactions. SLIT works by gradually exposing the patient’s immune system to the allergen, promoting greater tolerance and decreasing the severity of reactions. These results are significant as they indicate that SLIT may not only treat Hymenoptera allergies but also pave the way for the safe use of BV in therapies, minimizing the risks associated with traditional methods. Thus, the study highlights the need for further research to explore these therapeutic applications [150].

The myorelaxant and analgesic effects of BV ointment were evaluated in a study conducted by Nitecka-Buchta et al. [151] involving patients with temporomandibular joint dysfunction, classified according to the Research Diagnostic Criteria for Temporomandibular Disorders (RDC/TMD Ia and Ib). The patients were divided into two groups: 34 received the 0.0005% BV ointment, and 34 used a placebo. The results showed a significant reduction in muscle tension at rest and during maximal contraction in the masseter muscles of the BV-treated group, while the placebo group did not exhibit relevant changes. The VAS also indicated a more pronounced decrease in pain intensity in the BV group.

Table 2 presents studies that used BV as part of the therapeutic approach. We did not find clinical trials performed with BV, diabetes, and cancer. In Table 3, studies on neurodegenerative diseases have been performed.

Despite the growing body of experimental and early clinical evidence supporting the therapeutic potential of bee venom and its bioactive compounds, significant challenges remain that limit their broader clinical application. The same molecular properties responsible for the anti-inflammatory, anticancer, and immunomodulatory effects of bee venom may also contribute to cytotoxicity, allergenicity, and dose-dependent adverse reactions. In addition, variability in venom composition, differences in administration routes, and the absence of standardized formulations complicate reproducibility and translational interpretation across studies. Therefore, alongside its promising biomedical applications, careful consideration of bee venom’s safety profile, toxicological mechanisms, and pharmacokinetic limitations remains essential for the development of safe, clinically viable therapeutic strategies.

## 4. The Other Side of Bee Venom

Melittin, the main component of BV, is widely recognized for its therapeutic properties but also presents significant adverse effects. It is known to induce cell lysis, resulting in the death of healthy cells, and can provoke allergic reactions in about one-third of patients sensitive to BV. Additionally, melittin causes hemolysis, releasing hemoglobin from red blood cells, and has shown genotoxic potential, leading to DNA damage and alterations in gene expression associated with oxidative stress. Despite these negative effects, efforts are being made to mitigate melittin’s toxicity, such as introducing specific mutations that reduce hemolysis without compromising its antibacterial activity. The development of delivery techniques using nanocarriers also promises to facilitate the targeted administration of melittin, minimizing side effects on non-target cells and allowing its therapeutic properties to be explored more safely [152].

BV therapy can pose significant risks, including hepatotoxicity. Alqutub et al. [153] reported the case of a 35-year-old female patient with MS who underwent apitherapy with ten bees. After the treatment, the patient developed progressive jaundice, fatigue, and anorexia, lasting for three weeks. Clinical evaluation revealed predominantly direct hyperbilirubinemia, with serum bilirubin reaching 637 μmol and elevations in liver transaminases, although the liver appeared normal in size and showed no signs of structural damage. It is important to note that the patient gradually recovered, and the toxicity was reversible over eight weeks. These observations not only highlight the potential for hepatotoxicity associated with BV therapy but also underscore the need for additional systematic studies on the adverse manifestations of the venom across different patient profiles and health conditions to comprehensively assess its efficacy and toxicity.

The study conducted by Todorova et al. [154] provides evidence that BV induces the formation of superoxide anions and double-strand breaks in DNA in *Saccharomyces cerevisiae*, highlighting its cytotoxic and genotoxic potential. The authors state that “the cytotoxicity of BV can be attributed to its ability to cause direct damage to cell membranes, as well as to induce oxidative stress”. The research reveals that BV significantly increases oxidative stress at elevated concentrations, particularly in sensitive strains such as D7ts1. However, at controlled doses, components of BV, such as melittin and phospholipase A2, may activate immune responses and cellular repair mechanisms, suggesting therapeutic potential. While generally associated with adverse effects, the induction of DNA breaks could be explored in oncological contexts to sensitize tumor cells to treatment, underscoring the need for further investigation to elucidate the conditions that favor BV’s beneficial or detrimental effects. This complexity underscores the importance of a careful approach to the therapeutic use of venom, accounting for genetic individuality and dosage.

The article by Cavalcante et al. [14] analyzed the clinical complications of envenomation from bee stings, emphasizing the local and systemic reactions that may arise. Local reactions, such as intense pain, edema, and erythema, are often accompanied by severe systemic reactions. In the cardiovascular system, the venom can lead to arrhythmias and cardiogenic shock. The respiratory system is particularly vulnerable to anaphylaxis, leading to bronchospasm and glottic edema that can compromise breathing. The nervous system may present with symptoms such as headaches, seizures, and neuroparalytic effects. In the hematological system, complications such as disseminated intravascular coagulation and hemorrhages can occur. The renal system is also affected, with reports of acute renal failure linked to venom toxicity and rhabdomyolysis. The gastrointestinal system may also experience nausea and vomiting, while the musculoskeletal system can suffer from muscle pain and weakness.

**Figure 3:**
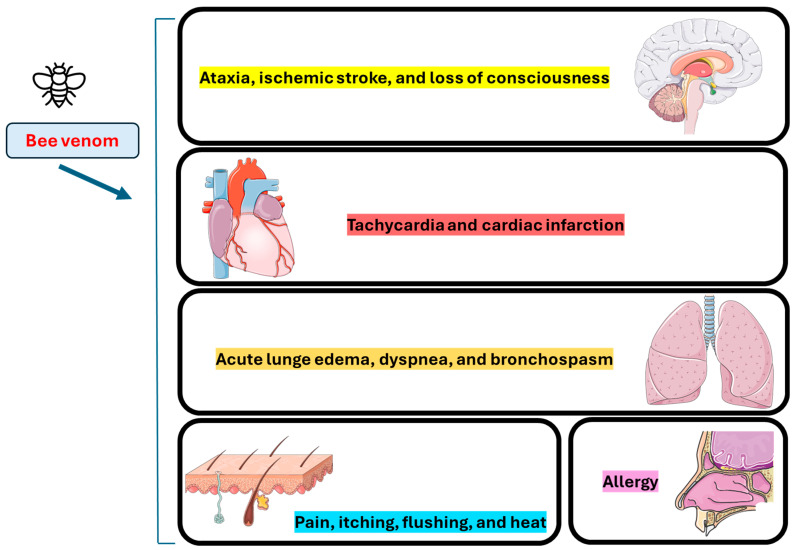
Representation of the local and systemic effects of bee venom on different organs. In the brain, it may cause ataxia, ischemic stroke, and loss of consciousness. In the respiratory system, acute edema, difficulty breathing, and bronchospasm can occur. In the heart, reactions include tachycardia and infarction. On the skin, symptoms include itching, flushing, heat sensation, pain, and papule formation. Allergic reactions are also common, highlighting the multifaceted impact of bee venom on the body.

Figure 3 shows the local and systemic effects of BV on different organs.

### Toxicity, Dose–Response Relationship, and Pharmacokinetic Challenges

**Figure 4:**
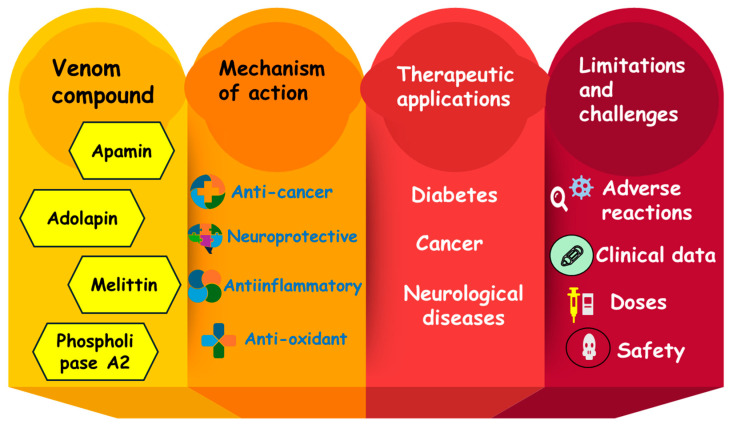
Bee Venom components, actions, and challenges in the use.

Despite the promising therapeutic potential of bee venom (BV), its clinical application is significantly constrained by toxicity concerns and an insufficiently characterized therapeutic window. BV exhibits a narrow margin between beneficial and harmful effects, largely due to the potent biological activity of its components, particularly melittin and phospholipase A2 (PLA2). Melittin, which constitutes approximately 40–60% of dry venom, is well known for its membrane-disruptive properties, leading to cell lysis and hemolysis at higher concentrations [20,21,22]. This dual behavior highlights the difficulty in defining a safe and effective therapeutic range. Figure 4 shows some challenges regarding the use of BV.

The dose–response relationship of BV is complex and often non-linear, with evidence suggesting a biphasic or hormetic effect. At low concentrations, BV and its constituents exert anti-inflammatory, antioxidant, and immunomodulatory effects, mediated through pathways such as NF-κB, MAPK, and Nrf2 [74,77,85]. However, as the dose increases, these beneficial effects are progressively replaced by cytotoxic effects, including membrane disruption, oxidative stress, and apoptosis in non-target cells [129,131]. This shift underscores the importance of precise dose optimization, which remains poorly standardized across studies.

Furthermore, variability in experimental design complicates the interpretation of dose–response relationships. Differences in venom composition—affected by bee species, geographic origin, and extraction methods—along with inconsistencies in administration routes and dosing units (e.g., μg/mL, mg/kg), limit reproducibility and cross-study comparisons [6,50]. In clinical contexts, individual variability, particularly related to immune sensitivity and allergic predisposition, further narrows the therapeutic window and increases the risk of adverse events, including anaphylaxis [42,43,44].

In addition to these challenges, the pharmacokinetic profile of BV remains inadequately understood. Key parameters such as absorption, distribution, metabolism, and excretion (ADME) have not been comprehensively characterized. BV peptides, including melittin and apamin, display high affinity for lipid membranes, facilitating rapid cellular interaction but also contributing to low selectivity and potential off-target toxicity [31,81]. These peptides are susceptible to enzymatic degradation; however, their rapid biological activity may precede metabolic inactivation, leading to acute cellular damage [84,87]. Data regarding their plasma half-life, tissue distribution, and elimination pathways are still scarce, particularly in human studies.

The therapeutic application of bee venom remains particularly challenging due to its narrow therapeutic window and pronounced dose-dependent toxicity. Melittin, which constitutes approximately 40–60% of dry bee venom, exhibits potent membrane-disruptive activity and may induce hemolysis, pore formation, mitochondrial dysfunction, and non-selective cytotoxicity at higher concentrations. Experimental studies have demonstrated that while low concentrations may exert anti-inflammatory or anticancer effects, higher systemic exposure can rapidly trigger tissue injury, severe inflammatory reactions, and destabilization of cellular membranes. In addition, toxicological responses may vary substantially according to the route of administration, including subcutaneous, intradermal, intravenous, or topical delivery, as well as individual susceptibility, allergic predisposition, and immune hypersensitivity [1,18,36].

Another major translational limitation involves the lack of standardization of bee venom preparations. Venom composition may vary across bee species, geographic origin, environmental conditions, seasonality, extraction methods, and storage conditions, thereby influencing the concentrations of bioactive compounds such as melittin, phospholipase A2, apamin, and hyaluronidase. This variability complicates reproducibility across studies and hinders accurate dose comparisons and pharmacokinetic interpretation. Furthermore, limited data are currently available regarding the absorption, biodistribution, metabolism, and long-term toxicity of bee venom-derived compounds in humans. Consequently, the development of standardized formulations, controlled purification methods, and targeted delivery systems represents a critical step toward safer and more reproducible therapeutic applications [1,36,155].

These pharmacokinetic limitations, combined with BV’s intrinsic toxicity, pose significant barriers to its clinical translation. Consequently, there is a pressing need for the development of advanced delivery systems, such as nanoparticle-based carriers, that can enhance target specificity, improve bioavailability, and reduce systemic toxicity [3,101]. Establishing standardized dosing protocols and conducting comprehensive pharmacokinetic and toxicological studies will be essential steps toward the safe and effective therapeutic use of bee venom.

## 5. Challenges and Translational Limitations of Whole Bee Venom-Based Therapies

Despite growing interest in the biomedical applications of BV, important challenges remain regarding its direct therapeutic use as a whole-venom preparation. Although BV exhibits anti-inflammatory, anticancer, neuroprotective, and metabolic effects in several experimental models, many of its major bioactive compounds, particularly melittin and PLA2, possess potent membrane-disruptive and cytotoxic properties that may compromise therapeutic safety. Melittin, which constitutes approximately 40–60% of dry bee venom, is an amphipathic peptide capable of inserting into lipid bilayers, promoting pore formation, membrane destabilization, hemolysis, mitochondrial dysfunction, and non-selective cytotoxicity at higher concentrations. Similarly, PLA2 hydrolyzes membrane phospholipids, generating inflammatory lipid mediators and contributing to membrane remodeling and cellular injury under certain conditions. These properties create a narrow therapeutic window in which beneficial biological effects may rapidly transition into systemic toxicity, depending on dose, route of administration, and individual susceptibility [1,156,157].

In addition to these toxicological concerns, allergic reactions and hypersensitivity responses remain important clinical limitations due to the strong immunogenic potential of several venom components. Collectively, these challenges reinforce the need for safer and more selective therapeutic strategies, including purified venom-derived compounds, peptide engineering, and targeted delivery approaches capable of preserving therapeutic efficacy while minimizing systemic adverse effects. Given these limitations, increasing attention has been directed toward the therapeutic investigation of isolated bee venom-derived molecules or engineered formulations rather than whole venom administration. Among these compounds, apamin has emerged as a particularly promising candidate due to its selective blockade of small-conductance Ca^2+^-activated potassium (SK) channels, allowing modulation of neuronal excitability and neuroinflammatory signaling with potentially greater specificity [158,159].

Similarly, melittin-derived conjugates, nanoparticle-based delivery systems, and targeted formulations have been investigated as strategies to preserve therapeutic efficacy while minimizing systemic toxicity and off-target membrane damage [160,161]. These approaches may improve bioavailability, tissue selectivity, and safety profiles, thereby enhancing translational feasibility.

Collectively, current evidence suggests that while whole bee venom possesses important biological activities, its clinical application remains limited by toxicological, pharmacological, and standardization challenges. Future research should prioritize the development of purified compounds, controlled formulations, and targeted delivery systems, alongside rigorous pharmacokinetic and toxicological evaluation, to establish safer and more reproducible therapeutic strategies based on bee venom-derived molecules.

## 6. Limitations of This Review

This review presents several limitations that should be acknowledged. First, the majority of available evidence on bee venom (BV) is derived from in vitro studies and animal models, limiting the direct translation of these findings into clinical practice. Human studies remain scarce, and when available, they often involve small sample sizes, short intervention periods, and heterogeneous methodologies.

Additionally, there is a lack of standardization in BV composition, dosage, and administration routes across studies. Variability in venom composition due to differences in bee species, geographic origin, and extraction methods further complicates comparisons and the reproducibility of results. This heterogeneity may significantly influence the biological activity and safety profile of BV.

Another important limitation is the insufficient characterization of the pharmacokinetics and pharmacodynamics of BV components, such as melittin and apamin. Data on absorption, distribution, metabolism, and excretion are still limited, which restricts a comprehensive understanding of their therapeutic window and potential toxicity.

Furthermore, adverse effects, including allergic reactions and systemic toxicity, are not consistently reported across studies, leading to an underestimation of potential risks. The lack of long-term safety data also represents a critical gap in the literature.

Finally, this review did not follow a systematic review protocol, which may introduce selection bias and limit the reproducibility of the search strategy and the inclusion criteria.

## 7. Future Perspectives

Future research on bee venom should prioritize the transition from preclinical findings to well-designed clinical trials with larger sample sizes, standardized protocols, and long-term follow-up. Establishing consensus on dosage, administration routes, and treatment regimens will be essential to ensure reproducibility and clinical applicability.

Advances in drug delivery systems, particularly nanotechnology-based carriers, represent a promising strategy to enhance the therapeutic efficacy of BV while minimizing its toxicity. Targeted delivery approaches may allow the selective action of bioactive compounds, such as melittin, on diseased tissues while sparing healthy cells.

Further investigations into the molecular mechanisms underlying BV activity are warranted, especially focusing on signaling pathways such as NF-κB, Nrf2, MAPK, and apoptotic cascades. Integrating omics technologies, including genomics, proteomics, and metabolomics, may provide deeper insights into the biological effects and variability of responses.

In addition, pharmacokinetic and toxicological studies are urgently needed to define safe therapeutic windows and better understand the metabolism and elimination of BV components. Personalized medicine approaches, including genetic profiling and genome-wide association studies (GWAS), may help identify patient populations more likely to benefit from BV-based therapies or at higher risk of adverse reactions.

Finally, future studies should also explore the sustainability and standardization of BV production, ensuring quality control and reproducibility, which are critical for its integration into evidence-based medicine.

## 8. Conclusions

BV presents a vast therapeutic potential, evidenced by its pharmacological properties, including anticancer, anti-inflammatory, antiviral, antioxidant, and neuroprotective effects. Investigations thus far demonstrate that the active components of BV, such as melittin, apamin, and phospholipase A2, exert beneficial effects across a range of health conditions, including neurodegenerative diseases and chronic inflammation, as well as cancer and diabetes. Furthermore, in clinical trials, the use of BV demonstrates its ability to complement conventional therapies, particularly for pain relief and reduced inflammation.

While previous reviews have primarily focused on isolated biological effects or single-disease applications of bee venom, the present review advances the field by providing an integrated, multi-disease perspective that combines mechanistic insights, translational strategies, and clinical evidence, highlighting bee venom as a multi-target therapeutic approach in complex chronic disorders.

Despite its promising pharmacological properties, the therapeutic application of bee venom still faces important translational challenges. Major components such as melittin and phospholipase A2 exhibit cytotoxic and membrane-disruptive effects that may limit the safe use of crude venom formulations. In addition, variability in venom composition and the lack of standardization complicate reproducibility across studies. These limitations reinforce the need for safer approaches based on isolated bee venom-derived compounds and targeted delivery systems. Unlike previous reviews focused on single diseases, this review provides an integrated perspective that combines molecular mechanisms, translational strategies, and clinical evidence on bee venom therapeutics.

## Figures and Tables

**Table 1:** Some main bioactive compounds in bee venom.

Component	Molecular Group	Effect	% of Dry BV	Reference
Melittin	Peptide	Anticancer, antiangiogenic, antifungal, antimicrobial, anti-inflammatory, antiarthritic, antiatherosclerotic, antiviral, analgesic, antiarrhythmic, antifibrotic, antidiabetic, hemolytic, and antinociceptive.	40–60	[26,30,31,32]
Phospholipase A2	Enzyme	Immunomodulatory, anticancer, neuroprotective, anti-inflammatory, analgesic, healing, antiviral, antiparasitic, antibacterial, and allergenic.	10–12	[33,34,35]
Apamin	Peptide	Antifungal, anti-inflammatory, cardioprotective, antifibrotic, anticancer, antibacterial, neuroprotective, and antiatherosclerotic	1–3	[36,37,38,39,40]
Hyaluronidase	Enzyme	Increases drug absorption, anti-edematous, healing, fertilizing, angiogenic, and embryogenic.	1.5–2	[41,42,43,44]
Mast Cell Degranulating Peptide	Peptide	Mast cell degranulation, release of histamine at low concentrations, and anti-inflammatory activity at higher concentrations.	1–2	[45,46,47]
Adolapin	Peptide	Antioxidant, anti-apoptotic, anti-inflammatory, analgesic, and antipyretic.	1	[36,48,49,50]
Histamine	Biologic amine	Allergenic and pro-inflammatory.	0.5–2	[30,41]
Secapin	Peptide	Anti-fibrinolytic, antibacterial, and anti-elastolytic	0.5–2	[36,45,51]

% of Dry BV: percentage of dry bee venom (the percentage that each component represents in relation to the total dry weight of bee venom).

**Table 2:** Effects of bee venom preparations observed in trials performed in humans.

Study	Model/Country	Population	Intervention/Comparison	Outcomes	Side Effects
[146]	Randomized, controlled, single-blind clinical trial/Egypt	66 ♂ patients, aged 28–50 years, postoperative from indirect unilateral inguinal hernioplasty	Group A received low-intensity pulsed ultrasound with BV gel; Group B received low-intensity pulsed ultrasound with plain gel.	Significant improvements in VAS (77.2% in Group A vs. 45.25% in Group B), CRP (39.62% in Group A vs. 12.33% in Group B), and hip ROM (various improvements across flexion, extension, abduction, and adduction).	Participants reported negative effects such as itching, but no specific adverse events were detailed
[147]	Randomized, assessor-blinded, pilot clinical trial/South Korea	60 participants, 33–56years, with non-specific chronic neck pain, 20 in each group (BV and NSAIDs, Combined)	Group 1 received BV acupuncture, Group 2 received loxoprofen (NSAIDs, 60 mg/tab, 3 times a day for 3 weeks), and Group 3 received both BV and NSAIDs for the same duration.	Significant improvements in pain intensity and functional disability were observed in all groups, with the BV group showing the greatest reduction in pain intensity and disability index by week 4.	Mild skin reactions (pruritus, rash, swelling) and systemic symptoms (headache, generalized myalgia, nausea)
[148]	Randomized, controlled, double-blind, multicenter clinical trial/USA	538 patients with knee osteoarthritis, 220 ♂, 318 ♀, mean age 56.9 years	Patients were divided into two groups: G1 received BV intradermal injections (1500 mg weekly for 12 weeks), and G2 received control (histamine)	The BV group showed significant improvements in WOMAC pain and physical function scores (*p* < 0.05) compared to the control, with sustained effects observed 4 weeks post-treatment.	HBV group: Injection site reactions (swelling 4.2%, discoloration 2.8%, pruritus 2.2%, erythema 1.9%, urticaria 1.4%). Control group: No significant adverse events
[149]	Randomized, double-blinded, placebo-controlled, parallel-group study/South Korea	54 patients with CLBP, 27 ♂, 27 ♀, aged not specified.	Patients were separated into 2 groups: one received six sessions of BV acupuncture with NSAIDs, and the other received sham injection with NSAIDs over three weeks.	BV treatment improved bothersomeness, pain intensity, functional status, and quality of life in patients with CLBP.	BV group: mild itching (n = 4), headaches (n = 1), generalized myalgia (n = 1), and dizziness (n = 2). Placebo: headaches (n = 2).
[151]	Randomized, double-blind, placebo-controlled clinical trial/Poland	79 patients with RDC/TMD Ia and RDC/TMD Ib, 10 ♂, 69 ♀, aged 22–34 years (average = 23 years).	Patients were divided into two groups: 34 received 0.0005% BV ointment applied locally over masseter muscles for 14 days, and 34 received placebo (vaseline).	The BV group showed a significant reduction in muscle tension at rest and during maximal contraction (*p* = 0.000001) and a significant decrease in pain intensity on the VAS scale (*p* = 0.000002). The placebo group did not show statistically relevant changes.	No adverse events were reported.

Abbreviations: BV: Bee Venom; BREF: Batterie Rapide d’Évaluation Frontale; CLBP: Chronic Low Back Pain; CRP: C-Reactive Protein; LLRs: Large Local Reactions; NSAIDs: Non-Steroidal Anti-Inflammatory Drugs; RDC/TMD: Research Diagnostic Criteria for Temporomandibular Disorders; ROM: Range of Motion; USA: United States of America; VAS: Visual Analogue Scale; WOMAC: Western Ontario and McMaster Universities Osteoarthritis Index.

**Table 3:** Clinical trials showing the effects of BV in neurological diseases.

Study	Model/Country	Population	Intervention/Comparison	Outcomes	Side Effects
[143]	Randomized, double-blind, three-armed, placebo-controlled clinical trial/South Korea	73 patients with IPD (exact gender distribution not specified).	Subjects were separated into 3 groups: one received acupuncture and BVA, one received sham treatment, and the third received conventional treatment.	The active treatment group showed significant improvement in UPDRS part II + III scores, gait speed, PDQL, and BDI compared to baseline.	Mild pain, slight bleeding after acupuncture, and mild itchiness or swelling after BVA were reported; no serious adverse events noted.
[142]	Randomized, double-blind, placebo-controlled, parallel-group single-center trial/France	40 patients with PD, 20 ♂, 20 ♀, Hoehn & Yahr stages 1.5 to 3.	Subjects were separated into 2 groups: one received monthly BV injections (100 μg), and the other received placebo for 11 months.	No significant difference in UPDRS III scores in the “off” condition between the BV and placebo groups after 11 months (*p* = 0.68). Secondary outcomes showed non-significant improvements in BREF and MMS scores in the BV group.	No significant adverse events reported.
[145]	Prospective, open-label, controlled clinical trial/Korea	11 patients with IPD, 7 ♂, 4 ♀, with a mean age of 65.5 years, received a stable dose of antiparkinsonian medication for at least 4 weeks prior to the start of the study.	Patients received acupuncture and BVA twice a week for 12 weeks while maintaining conventional treatment.	Significant improvements in gait speed, PDQL score, UPDRS part II, UPDRS part III, and combined UPDRS parts II and III scores compared to post-conventional treatment assessments.	Mild redness or mild itching after BVA; no serious adverse events reported.
[65]	Randomized, controlled, assessor-blind clinical trial/South Korea	43 adults with IPD, 13 ♂, 30 ♀, mean age 58.40 ± 10.04 years, mean duration of PD 5.97 ± 3.71 years.	Participants were divided into three groups: acupuncture (n = 15), BVA (n = 14), and control (n = 14). Treatments were administered twice a week for 8 weeks.	The BVA group showed significant improvement in the UPDRS (total score, parts II and III), the Berg Balance Scale, and the time to walk 30 m. The acupuncture group showed significant improvement in the UPDRS (part III and total scores) and the BDI. The control group showed no significant changes.	No significant adverse events reported. One participant in the BVA group experienced itching and was eliminated from the study.
[144]	Randomized crossover trial/The Netherlands	26 patients with relapsing multiple sclerosis, aged 18–65 years, EDSS up to 6.5, with at least one documented relapse in the past year or a gadolinium-enhanced lesion on MRI. Sex was not specified.	BV therapy (20 stings, 3 times/week for 24 weeks) versus no treatment period (24 weeks). Groups crossed over after 24 weeks.	Primary: No significant reduction in the number of new gadolinium-enhanced lesions on MRI between treatment and control periods. Secondary: No significant differences in relapse rates, EDSS, MSFC, SF-36, AFQ, and FIS.	No significant adverse events reported.

Abbreviations: AFQ: Abbreviated Fatigue Questionnaire; BDI: Beck Depression Inventory; BVA: Bee Venom Acupuncture; EDSS: Expanded Disability Status Scale; FIS: Fatigue Impact Scale; IPD: Idiopathic Parkinson’s Disease; MRI: Magnetic Resonance Imaging; MSFC: Multiple Sclerosis Functional Composite; PD: Parkinson’s Disease; PDQL: Parkinson’s Disease Quality of Life Questionnaire; SF-36: 36-Item Short Form Health Survey; UPDRS: Unified Parkinson’s Disease Rating Scale.

## Data Availability

No new data were created or analyzed in this study. Data sharing is not applicable to this article.
